# Build Your Own Equiluminance Helmet

**DOI:** 10.1177/2041669517716467

**Published:** 2017-07-07

**Authors:** Salammbo Connolly, Denis Connolly, Anne Cleary, Laura Herman, Patrick Cavanagh

**Affiliations:** Lycée Paul Claudel d’Hulst, Paris, France; Connolly-Cleary, Paris, France; Department of Psychology, Princeton University, NJ, USA; Laboratoire Psychologie de la Perception, Université Paris Descartes, Paris, France; Psychological and Brain Sciences, Dartmouth College, Hanover, NH, USA

**Keywords:** Colour, contours/surfaces, face perception, light, lightness/brightness, object recognition, optic flow, perception, time perception, temporal processing

## Abstract

A wearable ‘helmet’ version of the S cone isolating technique was constructed to explore vision at equiluminance. For my high school summer science project, I visited parks and streets while wearing the helmet and report that the helmet appears to have captured the main properties described for the large-scale, more cumbersome stage version.

For my summer 2016 school project, I worked on an equiluminising helmet with the help of my parents (Denis Connolly and Anne Cleary, artists and originators of the Meta-perceptual helmets, http://www.connolly-cleary.com/Home/helmets.html), Laura Herman and Patrick Cavanagh. The helmet was a new version of a technique originally presented in 1988 at the Bristol ECVP (12 years before I was born) that explored what the world looked like in pure colour, without the dark and light contrasts, and the contours, motion and depth given by luminance (Cavanagh, Adelson, & Heard, 1992). This is not easy to achieve because the same cones that give us colour also let us see luminance. To do so, the information coming to the visual system from the L and M (red- and green-sensitive) cones was blocked using a bright yellow light covering the entire visual scene (e.g., Stiles, 1959; Wald, 1966). The L and M cones together provide luminance information, and the bright yellow drives them to saturation, so they no longer respond differentially to spatial variations that would normally activate them. With the L and M cones saturated, the only spatially varying signal came from the S cones (blue-sensitive) that support principally the blue–yellow opponent-colour pathway (e.g., Eisner & MacLeod, 1980). The visual information was also passed through a blue filter to limit variations that would stimulate the L and M cones (Figure 1). This version was installed in a theatre in Bristol with the observers separated from the stage by a curtain, made of a deep blue filter and a veil lit from the auditorium side with intense yellow light. The audience could see objects through this, but only in shades of equilumininous white and yellow (Figure 2). They could make out objects and people on the stage, but most observers saw the depth as flat, as if the scene were printed on the curtain. Motion slowed, often dramatically. A spinning wheel appeared to slow or even stop completely. Faces were difficult to recognise. Subsequently, this stage version was presented at the ChromaFest, MIT Media Lab in 1990 and at VSS, Florida in 2004.

**Figure 1.**
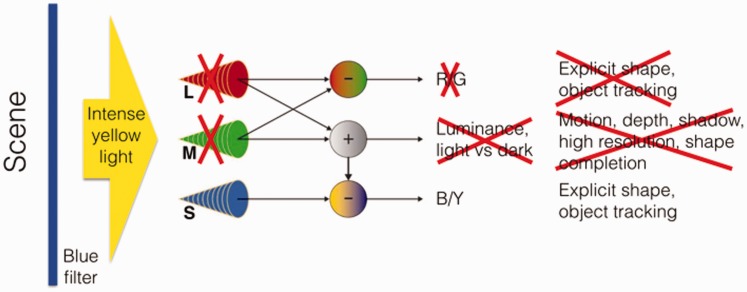
Diagram of the S cone isolating technique. The scene first passes through a deep blue filter and then an intense yellow is added that saturates the L and M (red and green sensitive) cones, disabling their contributions to motion, depth, shadow and fine detail. Only the S cones remain sensitive to spatial detail, and through the blue–yellow opponent pathway, they build a pure colour, white and yellow impression of the scene.

**Figure 2.**
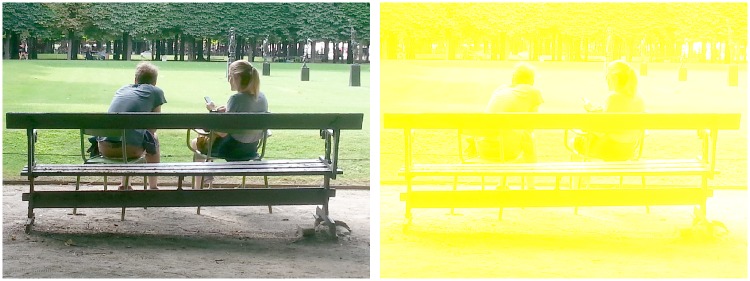
A scene in full colour and luminance and a simulation of the same scene in white and yellow, approximately as experienced through the equiluminance filter.

In our new project (Figure 3), we built a helmet using a miniature version of this technique. The first prototype (Figure 5) was adapted from a welder’s mask. The regular welding filter was replaced with one designed to behave like the equiluminising curtain of the stage versions. At the front, facing the scene, was a deep-blue filter (Lee 071). The filter had to cope with a range of lighting conditions, from artificial light to full sunlight, so we used two layers of deep blue film, the second as a flap that could be pulled down in sunlight. Behind this, we put an 8-mm-thick plexiglas panel, the surface scored with a 1 mm grid of lines (vertical lines on the front, horizontal lines on the back). Along the upper and lower edges of this panel, we installed LED strips (20 cm SMD-8020 LEDs 320-390 lm warm white) with yellow filters (Roscolux, Supergel R10), lighting the plexiglass so that the etched lines in the surface let out the internally reflected light and appear bright yellow, like the veil in the stage experiments (Figure 4).

**Figure 3.**
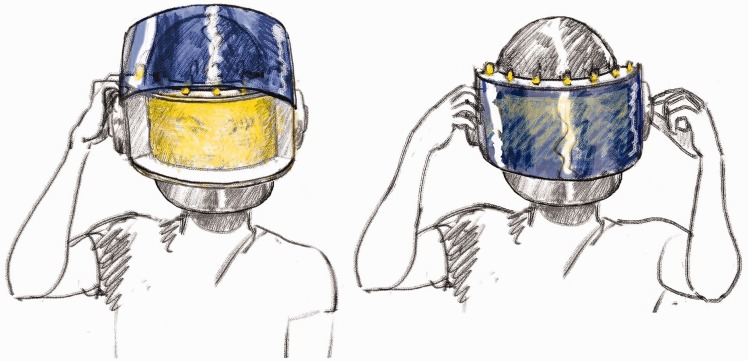
Early sketch of my parents’ design for the ‘equiluminance’ helmet.

**Figure 4.**
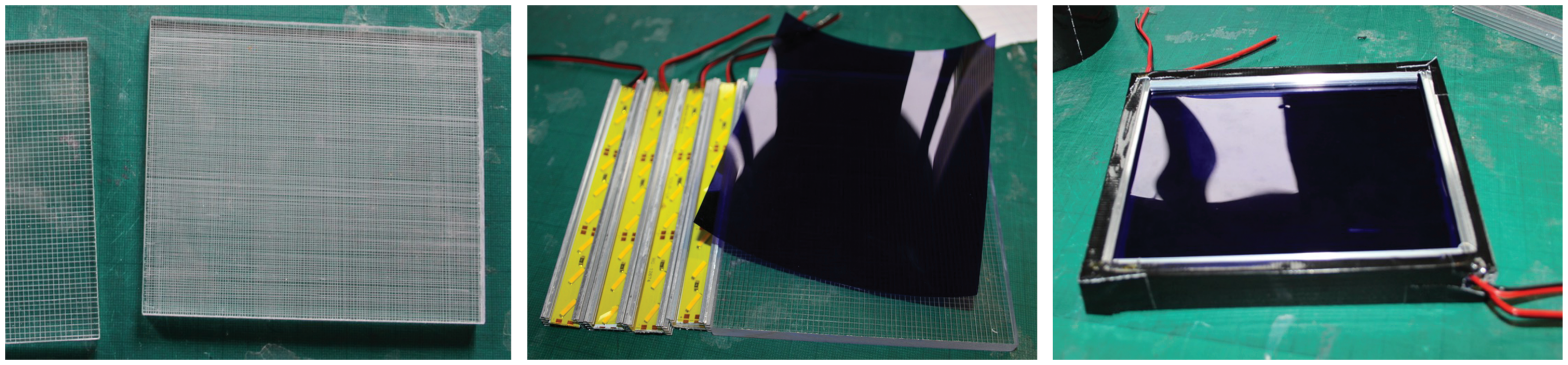
Left to right: etched plexiglass panel, yellow-filtered LED strips that side-light the panel, deep blue filter and the assembled equiluminising panel.

**Figure 5.**
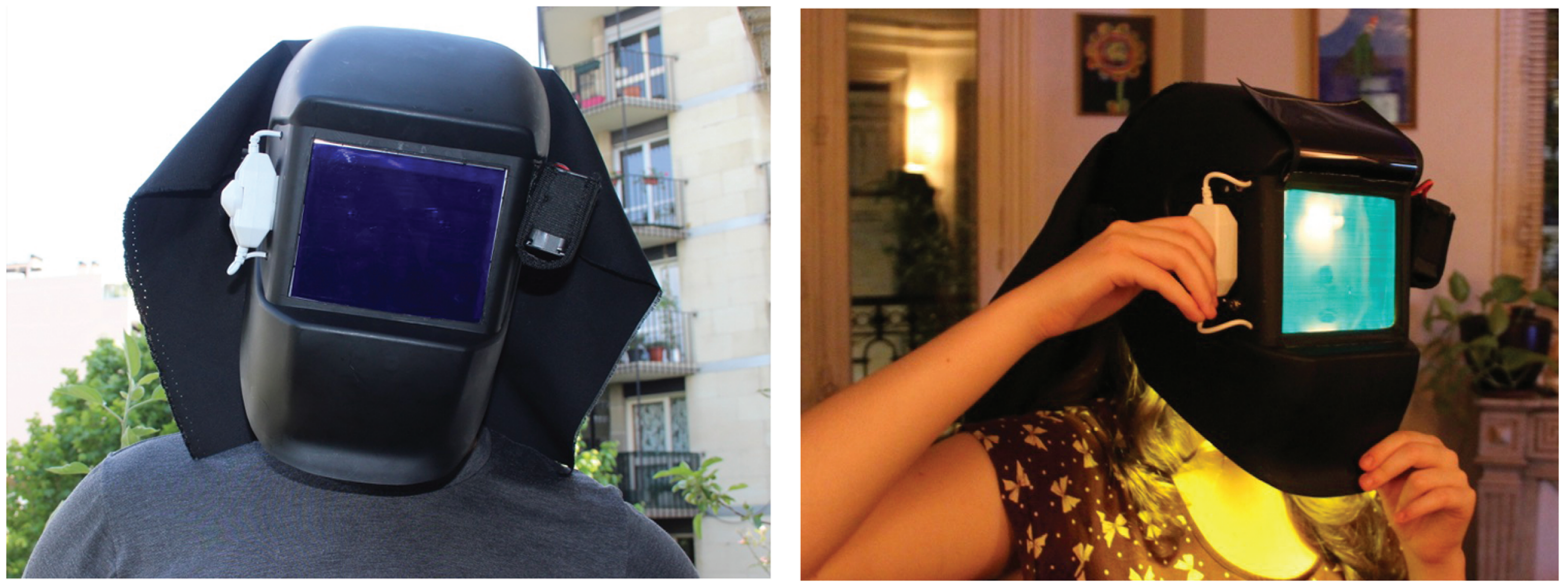
My father and I trying out the helmet, outdoors and indoors.

We placed a dimmer switch on the outside to allow users to vary the intensity of the light so that it just saturated the L and M cones, making motion slow and depth collapse. We added a battery pack with eight 1.5 V batteries in series to provide 12 V to drive the LEDs. To give extra lighting control, we added a switch to activate the top and bottom lights independently. The welder’s mask blocked the light from the front, other than that coming through the viewing screen, and we added a black shroud on the back to prevent light leaking in from behind (Figure 5).

In August 2016, we explored our neighbourhood wearing the prototype. It was extremely hot, which made the Darth Vader-like helmet quite uncomfortable. We stopped in squares and in parks, looking at buildings, at people, at cars and buses, at trees and benches. We attracted curious stares.

The city looked strange. Vehicles appeared to move sluggishly and then suddenly jerk forward. Shadows were deep and impenetrable, while bright things shone like gold. People we would normally recognise became strangers. A carrousel became a mirage of nodding horses and children. We looked at each other’s faces and they were like masks (Figure 6).

**Figure 6.**
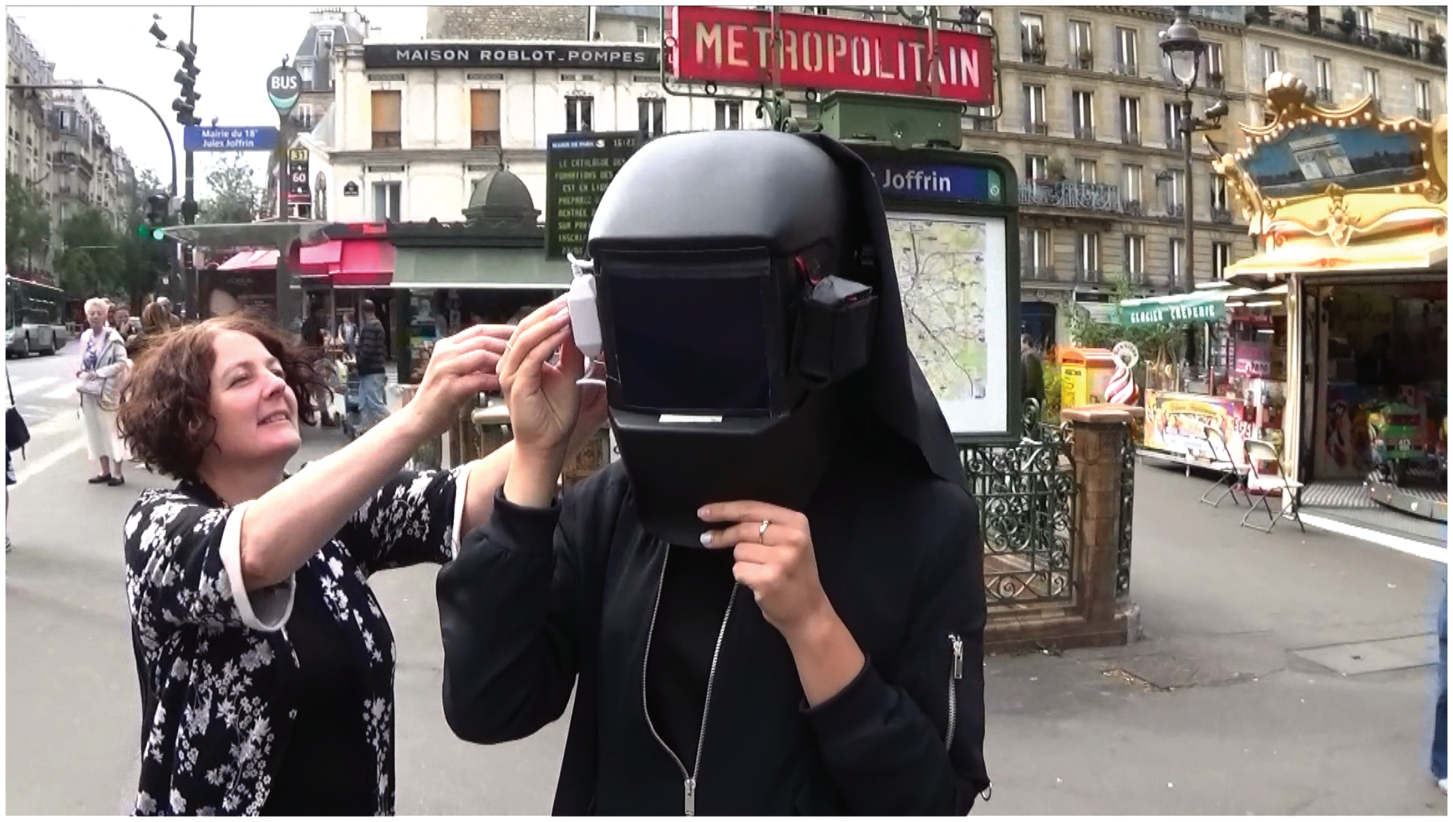
My mother and Laura Herman testing the prototype in Paris, August 2016.

We visited a park where I had played as a child. The helmet made this environment appear like a flat image projected on the little viewing screen, a remark that viewers at the earlier showings made as well, although there the screens were much larger. The trees seemed strangely metallic and the sky was pure yellow. The children seemed to play sluggishly, as if uninterested in their games. They surrounded us asking questions about the helmet. One of them wanted to try it on and we let him. But he seemed disappointed, explaining that he had expected the helmet to make him angry. We were puzzled. It was only later that we realised that he had understood ‘voir le monde en colère’ (see an angry world) instead of ‘voir le monde en couleur’ (see the world in colour)! Following these initial excursions, the helmet made a trip to Regensburg to be part of the Color meeting there in September 2016. Once the participants had secured the helmet on their head, they could regulate the yellow luminosity with the dimmer while watching several short videos. Several of these demonstrated that many illusions are preserved at equiluminance as long as the figure remained well resolved and involved connected elements. These observations had been noted previously at the MIT and VSS events and reported in a number of papers. For example, the Müller-Lyer and Poggendorf illusions are preserved, and structure from motion is seen for a rotating wire figure (Cavanagh, 1991; Li & Guo, 1995) but motion slows or stops (Cavanagh, Tyler, & Favreau, 1984), illusory contours and depth from shading are not seen and neither is structure from motion for rotating dot fields (Livingstone & Hubel, 1988). Hamburger, Hansen, and Gegenfurtner (2007) demonstrated the preservation of nine geometric-optical illusions under equiluminance as well as motion from some static figures (Hamburger, 2012). When using the equiluminising helmet, one particular video of a rotating wheel was critical for setting the intensity of the yellow light in order to view the other demonstrations. Participants were instructed to increase the intensity until the motion of the wheel slowed significantly, ensuring that equiluminance had been achieved. We did not record the level that each person chose and that would make a good experiment for future use of the helmets.

The helmet appears to have captured the main properties reported for the much larger stage versions. It could help to explore the properties of S cone-based vision in natural scenes and may be a useful addition to teaching and research projects at the university level concerning colour and vision at equiluminance (Gregory, 1977; Cavanagh, Anstis, & MacLeod, 1987).
